# Finding normal-to-better neurocognitive indexes in individuals with schizotypal traits using a social role task

**DOI:** 10.1038/s41537-023-00394-5

**Published:** 2023-09-29

**Authors:** Mingyi Diao, Ilya Demchenko, Gifty Asare, Jingyan Quan, J. Bruno Debruille

**Affiliations:** 1 Douglas Mental Health University Institute, Montreal, Qc Canada; 2 Department of Neurosciences, McGill University, Montreal, Qc Canada; 3 Department of Psychiatry, McGill University, Montreal, Qc Canada; 4 Department of Psychology, McGill University, Montreal, Qc Canada

**Keywords:** Schizophrenia, Neuroscience

## Abstract

Schizophrenia patients make more errors and have longer reaction times (RTs) than healthy controls in most cognitive tasks. Deficits are also observed in subclinical participants having high scores on the schizotypal personality questionnaire (SPQ). They are accompanied by smaller amplitudes of the event-related brain potentials (ERPs) that index attention and semantic- and working-memory. These functions are thus thought to be impaired in individuals having various schizophrenia attributes (SzAs). Nevertheless, normal RTs were recently found in SzAs during a particular self-referential task where half of the stimuli were names of extraordinary social roles (e.g., genius). Each name (ordinary or extraordinary) was presented individually, and participants were asked to decide whether or not they would consider themselves performing the role at any moment of their lives. To further test an absence of cognitive deficits in this task, the ERPs elicited by names of social roles were also examined in 175 healthy participants. The absence of longer RTs in high- than in low-SPQs was replicated. Moreover, the ERPs of high SPQs had larger occipital N1s, larger P2s and larger occipital N400s than those of low SPQs while late positive potentials (LPPs) were of similar amplitudes. Such results are consistent with clinical observations of greater attention and faster processing of stimuli related to extraordinary/delusional beliefs. Further studies should test whether the cognitive deficits found in SzAs are due to the use of tasks and stimuli that are less within their focus of interest than within that of healthy controls.

## Introduction

Schizophrenia patients often struggle to perform cognitive tasks, exhibiting lower accuracy rates and slower response times^1–3^. These cognitive deficits have a severe impact on their vocational and psychosocial rehabilitation^4–6^. Individuals with schizophrenia end up having lower income and higher unemployment rates^7,8^. The cognitive deficits of schizophrenia patients have thus been studied for many years.

Cognitive deficits are also found in subclinical people with schizophrenia attributes. Their performances in cognitive tasks resemble that of diagnosed schizophrenia patients^9^. Moreover, pharmacological studies have shown that antipsychotic medications induce comparable neurocognitive effects in individuals with high schizotypy and schizophrenia patients^10^. Schizotypy is thus gaining wider recognition as an “influential, comprehensive psychological construct in schizophrenia research”^11^. Measuring the degree of schizotypy can be used to locate each participant’s position on the normality to schizophrenia continuum^12,13^. Assessing schizotypal traits in the general population can also be used for early detection and preventive intervention in psychosis^14^. On the other hand, studying such subclinical participants circumvents the debilitating effects of disease chronicity and of previous exposure to antipsychotic medications. By employing the MATRICS Consensus Cognitive Battery (MCCB)^15^, numerous studies have shown that individuals with high schizotypy exhibit deficits of working memory^16,17^, attention^12,18^, executive functions^19^, incidental learning^20^ and have poor verbal IQ^21^. A meta-analysis of 67 studies confirmed that individuals with schizotypy have less verbal- and visuo-spatialworking memory^22^.

As could be expected, the event-related brain potentials (ERPs) evoked by the stimuli of the tasks used to study cognitive deficits of people with schizophrenia attributes (SzAs, i.e., schizophrenia patients and subclinical people with schizotypal traits) have consistently been found to be abnormal^23,24^. This is true for the amplitude (i.e., the voltage) of the occipital N1, a negative-going component peaking about 150 ms after the onset of the stimulus that indexes the amount of attentional resources allocated to the processing of the physical features of visual stimuli^25^. This amplitude has been found to be reduced in both schizophrenia patients^26,27^ and individuals with schizotypal traits^28^. This is also true for the amplitude of the P2, a positive ERP which peaks around 200 ms after the onset of the stimulus. It depends on the nature of the stimulus. For instance, it is larger and peaks earlier for pictures of faces than for written words. Its amplitude, which also depends on attention^29–31^, has been found to be smaller in schizophrenia patients than in healthy controls during cognitive tasks involving visual stimuli^32–34^.

The N400 ERP is also modulated. This negative-going ERP peaking at about 400 ms indexes semantic processing^35–37^. In schizophrenia patients, its amplitude to target words (e.g., butter) that are preceded by semantically unrelated words (e.g., socks) is generally found to be a bit smaller, and that to target words that are preceded by priming words (e.g., bread), a bit larger, than that of healthy controls, respectively. This double difference results in reduced N400 effects^38–41^. Healthy individuals having high scores at the Schizotypal Personality Questionnaire (the SPQ) also display reduced N400 amplitudes to target words that are semantically unrelated to priming stimuli^38,42^. Finally, abnormality in people with SzAs also pertains to the ERPs belonging to the family of late positive potentials (LPPs), such as the P300b of classical odd-ball protocols^43,44^, the P600 elicited by words^45,46^, and the so-called late positive component (the LPC)^47^. These large and late positive ERPs are well known to index the conscious evaluation of the stimulus^48,49^, working memory^50,51^, and the degree of emotion induced by the stimulus^52,53^. Patients with schizophrenia typically show reduced LPP amplitudes, which may serve as a neural marker of attentional dysfunction^54,55^. Several studies also reported reduced LPP amplitudes in subclinical participants with high scores at the SPQ, compared to individuals with low scores^56–58^.

In this context of deficits that seem to affect the processing of any stimulus in most cognitive tasks, the results of Fernandez-Cruz et al.^59^ appear to be particularly surprising. Indeed, the reaction times (RTs) of people with SzAs were normal despite the use of semantically rich stimuli and of a complex task requiring reflection. Namely, RTs were not observed to be longer than those of participants with no schizophrenia attributes. In that task, participants were presented with written names of social roles one by one and were asked to decide, as fast and as accurately as they could, whether or not they could consider playing the role at any moment of their lives. Half of these social roles were extraordinary. Among them, half were favorable (e.g., genius) and half of them unfavorable (e.g., killer). The unexpected finding of normal reaction times raises the possibility of an absence of cognitive deficits in tasks involving self-related choices and including stimuli related to extraordinary/delusional ideations/beliefs. Conversely, the abnormal reaction times observed with ordinary stimuli in self-*un*related tasks could be restricted to these tasks and to stimuli that are less within their focus of interest than within that of healthy controls. Such tasks and stimuli might not prevent the disengagement of people with SzAs, which might account for some degree of carelessness during cognitive tests^60^. Such stimulus material and self-*un*related choices might not capture their attention and/or motivate them as much as they motivate controls.

Clinicians usually observe greater interest in, heightened attention toward, and enhanced processing of, stimuli linked to the extraordinary/delusional ideations/beliefs of their patients. These frequent observations suggest that the cognitive deficits of people with SzAs could pertain only to meaning domains that are unrelated to these extraordinary domains. The aim of this study was thus to test the possibility of normal cognitive processes in these latter domains. As normal reaction times do not completely exclude the possibility of abnormal processes, we also examined well-established ERP measures (i.e., that of the N1, P2, N400 and LPPs) as indexes of the neurocognitive processes needed to perform the task. This rationale of our study could be important as confirming a normality of cognitive processes in the extraordinary domains of interest of SzAs could change therapeutic strategies. Cognitive deficits in ordinary domains could be treated by finding ways to raise their interest in these domains. The first goal of this study was to replicate the behavioral findings of Fernandez-Cruz et al.^59^. It was thus hypothesized that reaction times would not be longer in participants with high- than in participants with low-SPQ scores. Our second hypothesis was that ERP amplitudes would not be smaller in participants with high- than in participants with low-SPQ scores.

## Results

### Demographics

The demographics of the participants are shown in Table 1. Their SPQ scores covered a relatively wide range of the continuum between low and high schizotypy, namely, from 0 to 58 (out of 74, the maximal score). High schizotypy subgroup did not significantly differ from the low SPQ subgroup in terms of sex ratio, mean age, and mean number of years of education. The high SPQ group had a significantly lower mean score on the social desirability scale (the SDS) (F (1, 159) = 7.0, *p* = 0.009, *ηp*^2^ = 0.042). Compared to participants with lower SPQ scores, they were thus less likely to be biased in their acceptance of socially unfavorable roles and in their rejection of favorable ones.

**Table 1:** Demographic and clinical characteristics of participants.

		Low schizotypy	High schizotypy
		(*N* = 89)	(*N* = 86)
Sex: male	% (*N*)	43.8% (39.0)	51.2% (44.0)
Age	*M* (SD)	23.2 (3.1)	23.3 (3.1)
	Range	18.0–33.0	18.0–30.0
Years of education	*M* (SD)	14.8 (2.5)	14.3 (2.6)
	Range	1.0–18.0	2.0–18.0
Total SPQ	*M* (SD)	9.2 (5.6)	31.1 (9.3)
	Range	0.0–19.0	20.0–58.0
SDS	*M* (SD)	17.9 (5.6)	15.4 (4.9)
	Range	4.0–32.0	5.0–27.0

### Reaction times

**Fig. 1:**
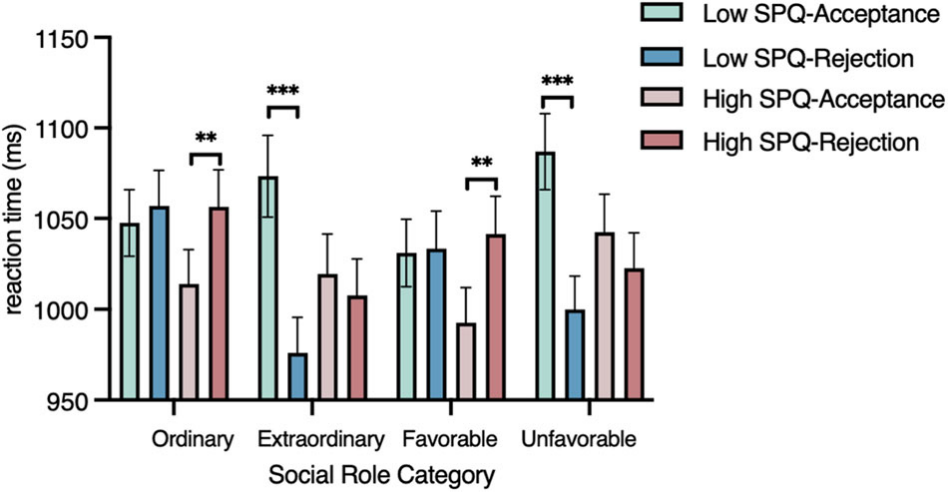
Mean reaction times for accepted- and rejected-social roles in each category in the low SPQ group (*N* = 89) and in the high SPQ group (*N* = 86). Thin bars display standard errors. ** are for *p* < 0.01. *** are for *p* < 0.001.

**Fig. 2:**
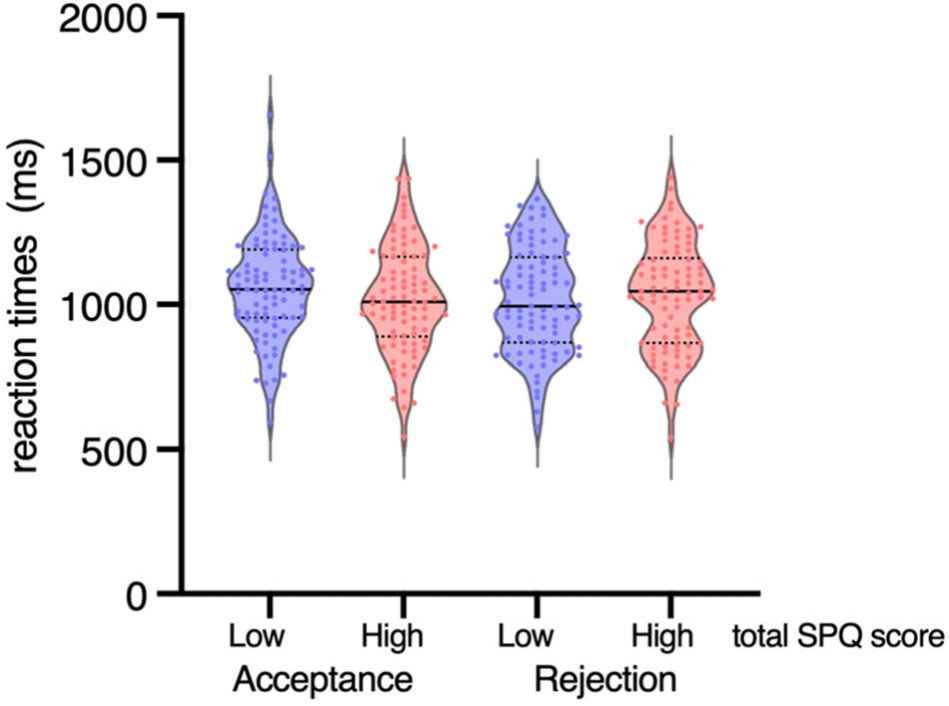
Reaction times for accepted- and rejected-social roles in the low SPQ group (*N* = 89) and in the high SPQ group (*N* = 86). Each dot represents each participant’s mean reaction time measure. The solid black lines are medians. The thin dotted black lines are quartiles.

The mean response times of the low- and the high-SPQ groups were 1038 ms (SD = 195) and 1024 ms (SD = 194), respectively. The mixed-ANOVA indicated a role category × decision × SPQ interaction (F (3, 519) = 3.6, *p* = 0.029, *ηp*^2^ = 0.021). Post hoc ANOVAs revealed that participants of the high SPQ group took longer to reject ordinary and favorable roles than to accept them. Conversely, participants with low SPQ scores took longer to accept extraordinary and unfavorable roles than to reject them (Figs. 1 and 2).

### Electrophysiological results

p* are *p* values judged significant according to the B-H FDR procedure, see Methods.

### N1 amplitudes

**Fig. 3:**
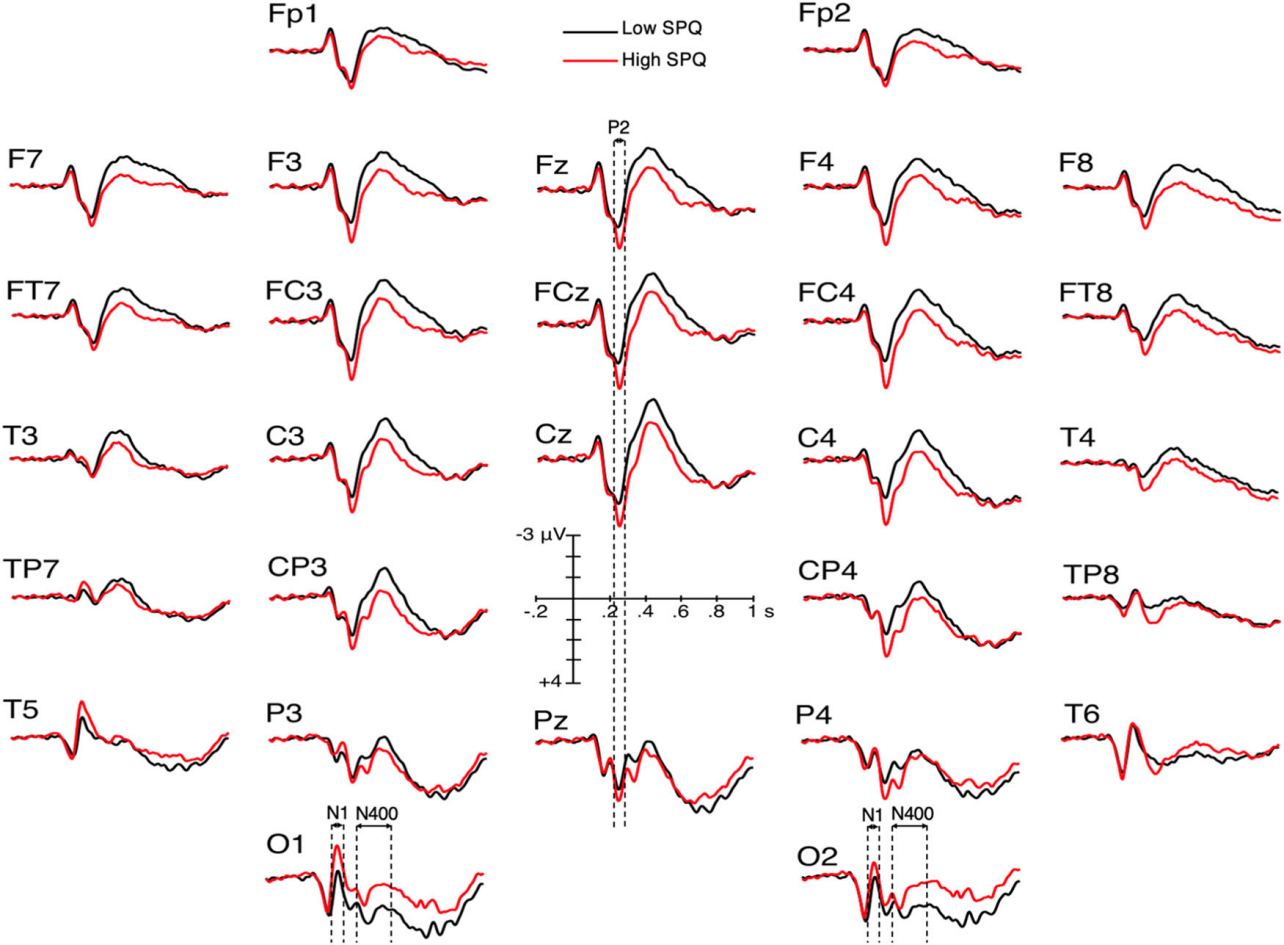
Grand average ERPs of the high SPQ group (*N* = 86, red lines) and of the low SPQ group (*N* = 89, black lines).

The ANOVA performed on N1 mean amplitudes revealed a significant SPQ × electrode interaction (F (27, 4671) = 3.4, p* = 0.023, *ηp*^2^ = 0.019). Post hoc ANOVAs revealed that high SPQ participants have larger N1 mean amplitudes at T5 (p* = 0.025), O2 (p* = 0.032) and O1 (p* = 0.007) than the low SPQ group (Fig. 3).

### P2 amplitudes

**Fig. 4:**
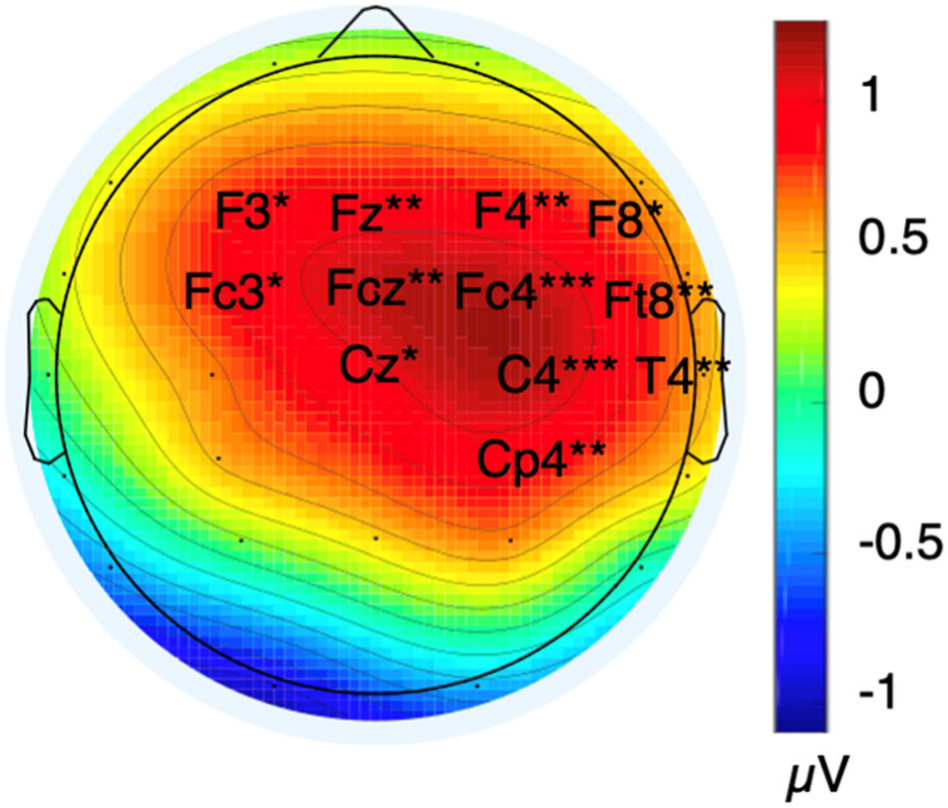
Spline interpolated iso-voltage scalp maps of the subtractions, at each electrode site, of the mean ERP voltages of the low SPQ group (*N* = 89) from those of the high SPQ group (*N* = 86) in the P2 (150–300 ms) time window. * are for 0.05 > *p* > 0.01. ** are for 0.01 > *p* > 0.001. *** are for 0.001 > *p*.

The ANOVA performed on P2 mean amplitudes revealed a significant SPQ × electrode interaction (F (27, 4671) = 4.5, p* = 0.004, *ηp*^2^ = 0.025). Post hoc ANOVAs revealed that high SPQ participants had larger P2 mean amplitudes than low SPQ ones at F8, Fz, Cz, T4, F4/3, Ft8, Fc4/3, Fcz, C4, and Cp4 (Fig. 4). The analyses designed to test the lateralization of the SPQ effect on P2 amplitudes (see Methods) revealed an antero-posterior location × SPQ interaction (F (6, 1038) = 5.6, p* = 0.008, *ηp*^2^ = 0.032) and an hemiscalp × SPQ interaction (F (1, 173) = 5.6, p* = 0.029, *ηp*^2^ = 0.027) for the parasagittal montage. We then focused on each antero-posterior location of this montage to look for the source of the hemiscalp × SPQ interaction. Post hoc ANOVAs revealed that P2 amplitudes were larger over the right- than over the left-hemiscalp for Fc4/3 (p* = 0.005), C4/3 (p* = 2.8 × 10^−4^), Cp4/3 (p* = 0.014) and P4/3 (p* = 6.6 × 10^−5^) only in participants with high SPQ scores. This was not the case for participants in the low SPQ subgroup.

On the other hand, role category interacted with electrode (F (27, 4644) = 3.8, p* = 0.003, *ηp*^2^ = 0.022). Ordinary roles were found to elicit larger P2s than extraordinary roles at P3 (p* = 0.038), T6 (p* = 0.001), T5 (p* = 0.027), O2 (p* = 3.4 × 10^−4^) and O1 (p* = 3.1 × 10^−4^).

### N400 amplitudes

N400 waves started from larger P2s for high- than for low-SPQ scorers (Fig. 3). We remedied this baseline problem by measuring N400 amplitudes from the P2 peak to the N400 peak in both groups. This was done in a region of interest (ROI) centered on the electrode at which the P2 was the largest, that is, Cz. This ROI included Cz, C3, C4 and Fcz. The voltage of the peak of the P2 at each of these ROI electrodes was averaged and compared with the average of the voltage of the peak of the N400 at these ROI electrodes. Making these measures on grand averages revealed that the N400s of high SPQ scorers were very slightly larger than those of low SPQ scorers (i.e., 4.16 vs. 4.08 μV, respectively). Such a small difference could not be significant. No analysis was thus performed. The amplitudes of the N400s of high SPQ scorers were similar to those of low SPQ scorers in this ROI. Nevertheless, at occipital sites (O1/2), the mean amplitudes of N400s appeared larger for the high- than for the low-SPQ group (see Fig. 3). The analysis run there revealed an effect of SPQ (F (1, 173) = 4.6, p* = 0.033, *ηp*^2^ = 0.026).

**Fig. 5:**
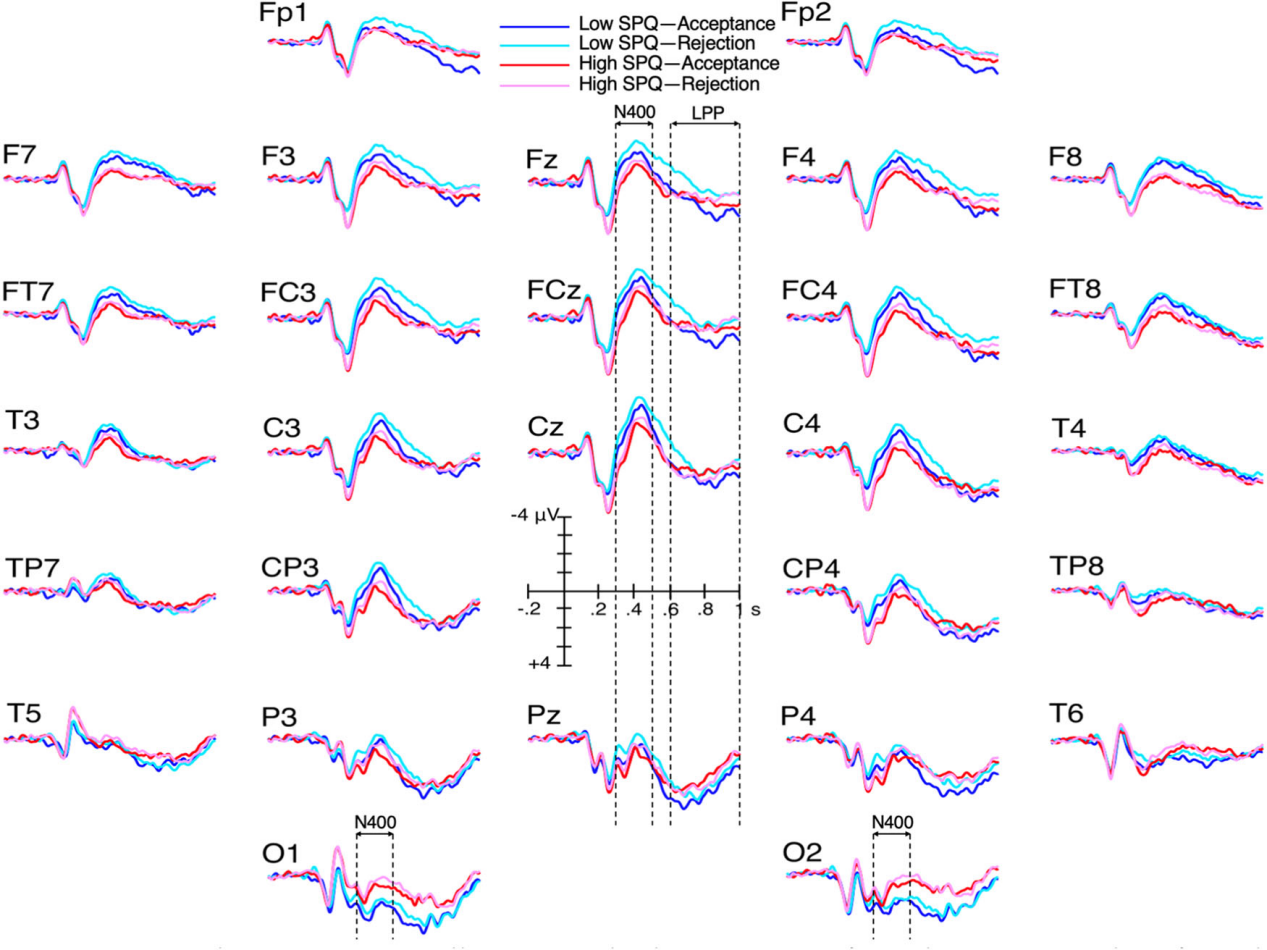
Grand average ERPs illustrating the larger N400s for role rejections than for role acceptances. ERPs of participants of the low SPQ group (*N* = 89) are in light blue lines for rejections and dark blue lines for acceptances. For the high SPQ group (*N* = 86), the light red lines are for rejections, and the dark red lines for acceptances.

**Fig. 6:**
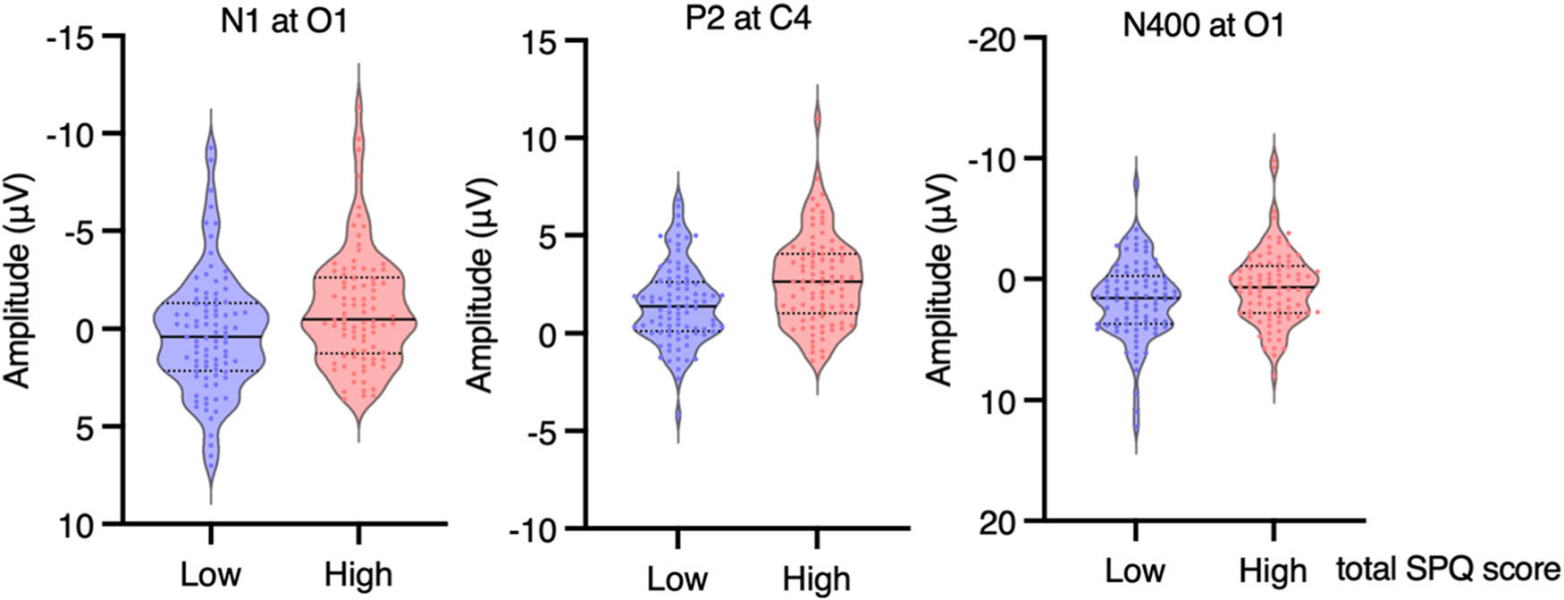
Amplitudes of N1s at O1, of P2s at C4 and of N400s at O2 for the low SPQ group (*N* = 89, blue plot) and of the high SPQ group (*N* = 86, red plot). Each dot represents each participant’s mean voltage measure. The solid black lines are medians. The thin dotted black lines are quartiles.

On the other hand, decision interacted with electrode (F (27, 4671) = 2.8, p* = 0.014, *ηp*^2^ = 0.016). Post hoc ANOVAs revealed larger N400 amplitudes at all electrodes for social role rejections than for acceptances (Fig. 5). Finally, there was a category × electrode interaction (F (27, 4671) = 2.2, p* = 0.058, *ηp*^2^ = 0.013) (see Supplementary Table). N400 amplitudes were larger for extraordinary- than for ordinary-roles at F3, C3/4, Cp3/4, P3/4, O1/2, Cz, Pz, F7, Ft7/8, T3/4, Tp8, and T5/6. Figure 6 displays the mean amplitudes of the N1, P2, and N400 time windows, respectively, at the electrode exhibiting the most significant SPQ effect.

### LPP amplitudes

There was no main effect of SPQ nor any interaction that included SPQ for LPP measures. LPPs of high SPQ scorers were of amplitudes similar to those of low SPQ scorers. On the other hand, a decision × electrode (F (27, 4671) = 7.4, p* = 4.25 × 10^−7^, *ηp*^2^ = 0.041) and a category × electrode interaction (F (27, 4671) = 8.3, p* = 3.14 × 10^−8^, *ηp*^2^ = 0.046) were found in this time window. LPP amplitudes were larger when participants were accepting roles than when they were rejecting them at Fp2/1, Fz, F4/3, Fc4/3, and Fcz. However, the opposite result was found at T5 (p* = 0.047). Larger LPPs were also found for extraordinary- than for ordinary-social roles at Fp2/1, Pz, P4/3, T6/5, C4, Tp8/7, Cp4/3, and O2/1.

### Correlations between clinical scores and role acceptance percentages

As illustrated in Table 2a, the strongest positive correlation between the acceptance rates of roles and total SPQ scores was for extraordinary roles (*r* = 0.32, *p* = 9.0 × 10^−6^*, R*^2^ = 0.10), which replicated Fernandez-Cruz et al.’s finding^59^. This correlation was seen with the delusion (*r* = 0.34, *p* = 3.0 × 10^−6^*, R*^2^ = 0.12), disorganization (*r* = 0.29, *p* = 4.1 × 10^−5^, *R*^2^ = 0.08), and interpersonal factor (*r* = 0.20, *p* = 4.0 × 10^−3^, *R*^2^ = 0.04) of schizotypy. The correlation between the acceptance rates of extraordinary roles and the Peters et al. Delusion Inventory (PDI) total numbers of “yes” was also noticeable (*r* = 0.40, *p* = 1.6 × 10^−8^, *R*^2^ = 0.16). With combined role categories (Table 2b), similar patterns were found between the rates of acceptance of extraordinary unfavorable roles and clinical scores. The strongest correlation was with the PDI Total Yes score (*r* = 0.44, *p* = 7.8 × 10^−10^, *R*^2^ = 0.19), followed by that with the total SPQ (*r* = 0.37, *p* = 2.9 × 10^−7^, *R*^2^ = 0.14), delusion (*r* = 0.36, *p* = 5.4 × 10^−7^, *R*^2^ = 0.13) and disorganization scores (*r* = 0.35, *p* = 1.0 × 10^−6^, *R*^2^ = 0.12). Additionally, the total SPQ score was strongly correlated with the total role acceptance percentage (all role categories combined) (*r* = 0.25, *p* = 3.4 × 10^−4^, *R*^2^ = 0.06).

**Table 2:** Correlations between clinical scores and role acceptance percentages.

a. Pearson’s correlation coefficients between the percentages of acceptance for each social role category and clinical scores								
Clinical scores	Ordinary roles		Extraordinary roles		Favorable roles		Unfavorable roles	
	*r*	*p*	*r*	*p*	*r*	*p*	*r*	*p*
Total SPQ	0.10	0.086	0.32	9.0 × 10^−6^	0.17	0.011	0.31	2.1 × 10^−5^
SPQ Interpersonal	0.04	0.32	0.20	0.004	0.07	0.16	0.20	0.004
SPQ Delusion-like ideation	0.13	0.048	0.34	3.0 × 10^−6^	0.23	0.001	0.29	6.2 × 10^−5^
SPQ Disorganization	0.12	0.050	0.29	4.1 × 10^−5^	0.16	0.018	0.31	1.4 × 10^−5^
Total PDI Yes	0.17	0.015	0.40	1.6 × 10^−8^	0.28	1.1 × 10^−4^	0.35	8.0 × 10^−7^
Total PDI	0.09	0.11	0.32	8.0 × 10^−6^	0.20	0.004	0.27	1.9 × 10^−4^
PDI Distress	0.07	0.19	0.25	4.5 × 10^−4^	0.14	0.035	0.22	0.002
PDI Preoccupation	0.11	0.08	0.33	3.0 × 10^−6^	0.22	0.002	0.27	1.7 × 10^−4^
PDI Conviction	0.09	0.13	0.32	8.0 × 10^−6^	0.20	0.005	0.26	2.5 × 10^−4^

b. Pearson’s correlation coefficients between the percentages of acceptance for each social role category combination and clinical scores								
Clinical scores	Ordinary favorable roles		Extraordinary favorable roles		Ordinary unfavorable roles		Extraordinary unfavorable roles	
	*r*	*p*	*r*	*p*	*r*	*p*	*r*	*p*
Total SPQ	0.01	0.46	0.25	3.8 × 10^−4^	0.17	0.012	0.37	2.9 × 10^−7^
SPQ Interpersonal	−0.05	0.26	0.15	0.027	0.11	0.078	0.25	4.0 × 10^−4^
SPQ Delusion-like ideation	0.07	0.17	0.29	6.4 × 10^−5^	0.15	0.025	0.36	5.4 × 10^−7^
SPQ Disorganization	0.01	0.43	0.23	0.001	0.20	0.004	0.35	1.0 × 10^−6^
Total PDI Yes	0.10	0.095	0.34	2.0 × 10^−6^	0.19	0.006	0.44	7.8 × 10^−10^
Total PDI	0.02	0.38	0.28	8.4 × 10^−5^	0.14	0.032	0.33	4.0 × 10^−6^
PDI Distress	−0.01	0.44	0.21	0.002	0.13	0.049	0.26	2.1 × 10^−4^
PDI Preoccupation	0.04	0.30	0.30	2.8 × 10^−5^	0.14	0.030	0.33	3.0 × 10^−6^
PDI Conviction	0.02	0.42	0.28	7.0 × 10^−5^	0.14	0.036	0.33	5.0 × 10^−6^

## Discussion

This study replicated the behavioral results obtained in the social role acceptance task used by Fernandez-Cruz et al.^59^. Reaction times were not longer in participants with high- (*N* = 86) than in participants with low-SPQ scores (*N* = 89), confirming the absence of suboptimal cognitive functioning in that task. Moreover, the N1, P2, N400 and LPP ERPs of high SPQ scorers were not of smaller amplitudes than those of low SPQ scorers, supporting an absence of deficit of attention, semantic processes and working memory. In fact, their occipital N1s and central P2s were found to be of significantly larger amplitudes, as well as their N400s at occipital sites. These results thus support not only an absence of cognitive deficits in high SPQ participants in this particular task. They also indicate that this task might be more attractive to them than for the low SPQ scorers. Indeed, early ERPs indicate high SPQ scorers allocated a bit more attention to the task. These results either mean that people with schizophrenia attributes do not really have a general cognitive deficit, or that they do have such a deficit but that it could be compensated for in certain domains, such as that of the extraordinary.

The cognitive deficits observed in people with schizophrenia attributes (SzAs, i.e., schizophrenia patients and subclinical people with schizotypal traits) in other tasks could be, at least in part, due to the use of more ordinary stimuli and of self-*un*related tasks. In this regard, some previous self-referential studies have reported that people take less time to respond to self-related- than to self-*un*related-conditions when asked to decide if personal trait adjectives described their own or others’ personality^61,62^. This effect could be greater in high SPQs. They might have involved their selves to an even greater extent than low SPQs. However, Holt et al.^63^ revealed dysfunction of the default network in patients with schizophrenia during self-referential tasks. Notwithstanding apparent similarities, it is nevertheless needed to mention a salient disparity between self-referential paradigms and the present social role task. In the latter, participants are asked to imagine if they would play various social roles. This diverges from the mere descriptive exercise of deciding whether or not an adjective, such as, “honest”, describes them. Moreover, extraordinary social roles tap into grandiose or delusional thinking which may then result in both specific behavioral and ERP effects.

Larger occipital N1s signal that more attentional resources were allocated to the processing of the visual features of the stimuli in high- than in low-SPQ scorers. Larger P2s could also be due to this additional amount of attention. However, they might also be related to the fact that the stimuli and the task were self-relevant. Previous research has shown that larger P2 amplitudes are elicited by stimuli that are more self-relevant^34,64^. Globally, high SPQ scorers accepted a larger number of roles than low SPQ scorers, thus showing that they found more roles to be self-relevant, consistent with their lower self-concept clarity^65^, which may be associated with fewer incompatibilities. On the other hand, the high acceptance rates of roles in high SPQs may also reflect their openness to new experiences, sensation-seeking and creativity^66^. Previous studies have suggested that creativity is associated with psychoticism, positive schizotypy^67^, and the right hemisphere^68,69^. Observing larger P2s over the right- than left-hemiscalp in high SPQs could thus also be related to their greater creativity.

Larger N400 amplitudes for rejected- than for accepted-roles were observed, consistent with Metzler et al.’s finding^62^ that self-*in*congruent information elicits more negative N400 amplitudes than self-congruent stimuli. Incidentally, that role names of preceding trials could have primed the name of the current trial was unlikely. Each role had to be responded to for itself, not taking into account the previous role. Moreover, the order of the social roles in each of the two subsets was chosen by a randomizing procedure, and roles were, in the vast majority of cases, not semantically related to each other, as can be seen in their list, which has been placed in the Supplementary Materials.

Interestingly, N400s of high SPQ scorers were found to be of significantly larger amplitudes than those of low SPQ scorers over occipital sites. The allocation of a greater amount of attentional resources to semantic processes correlates with larger N400 amplitudes^70^. However, ERPs at these sites typically pertain to the processing of visual information. Nevertheless, these N400 effects could be a direct, or an indirect, index of an activation of the imagery corresponding to the meanings of social roles. According to embodied theories of cognition (e.g., Matheson et al.^71^), such activations are integral to the coding of the meaning of imageable concepts, of which social roles are part and parcel (for the effects of such visual semantic information on posterior N400, see Kellenbach et al.^72^). For instance, the presentation of the social role name “Olympic Champion” can automatically activate, among other things, the visual representation of the three champions receiving their medal on the podium. When high SPQ scorers are asked whether or not they could consider playing this role, they might be more likely to image themselves (i.e., to picture themselves) playing it than low SPQ scorers. The larger N400 amplitudes observed over occipital sites in high- than in low-SPQ scorers thus suggest a greater allocation of attentional resources for the processing of the visual representations related to the meaning of the roles.

The important absence of interaction of group with role condition on N400 amplitudes at occipital sites suggests that such image processing was boosted in a sustained way throughout the task for every role in high SPQ scorers. Thus, instead of a mere capture of their attention by each extraordinary role, these roles might have boosted a general interest for the task, which could be due, at least partly, to the (justified) expectations of upcoming extraordinary roles.

In conclusion, it seems important to mention again that ERP results are in sharp contrast with previous ERP findings, in which smaller ERPs were found in SzAs than in healthy controls, as summarized in the introduction. This could be due to the nature of the social role task used, which involved self-related choices, and to extraordinary roles. This task may have given participants with high SPQ scores the opportunity to express the variety of their social drives. This may have made the task more attractive and motivating for them than for low SPQ scorers^73,74^. These surprising results are in fact consistent with clinical observations that patients with schizophrenia tend to pay more attention to, and process more quickly, materials that may be associated with delusional beliefs/ideations.

Among the limitations of the study, one might include the fact that low- and high-SPQs were simply teased apart by a median split, which wastes information about their precise SPQ scores. However, it allows to have two grand average ERPs and thus to see the global shape of the differences. Another limitation of the study is that our participants were not schizophrenia patients. The findings reported here might thus be observed only when symptoms are not severe and when there is a lack of dysfunctions that could be specific to patients^75^. For instance, the dysfunctions induced by chronicity, social isolation, lack of challenges when having no job and major tranquilizing effects of antipsychotic medications. Future studies should thus use this social role task in patients with schizophrenia. Such studies could also consider adding conditions where participants would be asked to decide whether someone they are familiar with could consider playing the roles, in order to explore the difference between self- and other-perspective processing.

Finally, it seems that the neurocognitive indexes found in the social role task should have been compared to those obtained in a control matching task for the participant sample used. However, finding such a control task was thought to be too hazardous. Indeed, too many tasks and stimuli would have to be tried before finding the one that matches the physical and semantic characteristics of the names of social roles used, the degree of difficulty of the social task and the brain regions involved^76^ so that the sizes of raw ERPs at each scalp site and the schizotypy effects can actually be compared across the two tasks. This is why there was no control task in this first study of the ERPs elicited by names of social roles in the acceptance/rejection task. Nevertheless, a large number of participants (175) was used and selection criteria were similar to those of many schizotypy studies that found poorer behavioral performances and smaller ERPs in high- than in low-SPQs using a variety of tasks and stimuli. In this context, normal behavioral performances and better ERPs in high- than in low-SPQs needed to be reported, just as lower performances and smaller ERPs in high SPQs were initially reported in the literature.

## Methods

### Participants

One hundred and ninety-two participants between the ages of 18 and 33 were recruited through online advertisements in both English and French posted on a variety of social media (e.g., Kijiji, Facebook, and the McGill Classified Ads website). Participants were 174 English and 18 French mother-tongued with at least 10 years of education in either language and normal or corrected-to-normal vision. The experiments proceeded in their mother language. They reported having no previous history of neurological medical conditions that compromise brain functioning, and no substance abuse, intellectual deficits, or intake of medications for a psychiatric disorder during the two previous years. All participants gave written informed consent forms prior to participation and the data were processed anonymously. This study was approved by the Douglas Ethics Review Board (project number: IUSMD-06-42). All research was performed in accordance with relevant guidelines/regulations and in accordance with the Declaration of Helsinki.

### Psychometric scales

A demographics form, the schizotypal personality questionnaire (SPQ), the 21-item Peters et al. Delusions Inventory (PDI) and the Social Desirability Scale (SDS) were filled out by every participant before the testing. The SPQ is a questionnaire that was initially designed to measure the severity of schizotypal personality traits in the general population^77^. Nevertheless, it is based on the DSM-III-R criteria used to diagnose full-blown schizophrenia^78–80^. It is a self-report questionnaire that contains seventy-four items. It has high internal reliability (alpha >0.90) and test-retest reliability (*r* = 0.82). The three main factors of the SPQ are delusion, interpersonal deficits, and disorganization, in both males and females and in both healthy adolescents and young adults^81,82^. The PDI is a questionnaire measuring delusional ideation in the general population^83^. It has good internal consistency (i.e., from 0.52 to 0.94) and a test-retest reliability between 0.78 and 0.8. For each delusional idea, participants are asked to answer “YES”/ “NO” to indicate whether they have it and, if they do, express their associated levels of distress, preoccupation, and conviction on a Likert scale ranging from 1 to 5. The total “Yes” score ranges from 0 to 21 and the total score for the entire test is from 0 to 105. Finally, the social desirability questionnaire (SDS) includes 33 items whose answers were used to evaluate the tendency that could make participants respond in a more socially desirable manner^84^, biasing the decisions to accept certain roles and to reject others. For example, individuals who strongly desire social approval may be more inclined to conceal some liked roles and accept favorable roles to make them look better to the researcher or to avoid appearing critical of roles endorsed by the majority of the population.

### Stimuli

The social role acceptance task of Fernandez-Cruz et al.^59^ was used. It has both an English and a French version. Two subsets of 200 names of social roles were extracted from the set of 401 names used in Fernandez et al.^59^. They included the same number of roles of each of the four categories (i.e., ordinary favorable, ordinary unfavorable, extraordinary favorable and extraordinary unfavorable). For the purpose of having a brief experiment, only one 200-word subset was presented to each participant. This particular subset was chosen at random. Statistical analysis of the data derived from Google Books Ngram viewer revealed no significant differences in the mean numbers of letters and the mean frequencies of use across the stimulus conditions (see Supplementary Materials).

### Procedure

Upon arrival, participants completed a demographics questionnaire where they provided information regarding their sex, age, and level of education. Immediately after completing the set of psychometric scales, the EEG recording session began. Participants were seated comfortably in a dimly lit room. Their eyes were roughly 70 cm away from the computer screen. The names of roles, written in black 20-point font on a white background, were presented one at a time in several different random orders across participants. At each trial, participants were asked to decide as quickly as possible if they would consider performing the role at any moment of his/her life. The index and middle fingers were used to make a “YES” or “NO” decision, respectively. For instance, when participants were presented with the social role name of “teacher”, they had to decide whether they could consider being a teacher at any given point in their lives. Presentation times and delays were jittered to prevent the development of contingent negative variations (see Supplementary Materials). Each role was presented for at least 500 ms, and at most 1800 ms, immediately followed either by a fixation cross which lasted for 300 to 2500 ms or by a “BLINK!” stimulus which lasted for 500 to 1000 ms.

### Data acquisition

The time (RT) taken to decide whether to accept or reject the role of each trial was recorded. An Electro-Cap International (ECI) cap including 28 tin electrodes was used to record the electroencephalogram (EEG) at the Fp1/2, F3/4, Fc3/4, C3/4, Cp3/4, P3/4, O1/2, Fz, Fcz, Cz, Pz, F7/8, Ft7/8, T3/4, Tp7/8, and T5/6 sites of the international 10-20 system. The right earlobe was used as the reference. The ground was placed 2 cm anterior to Fz. Impedances were kept below 5 KΩ. An electronic notch filter was used to reduce the 60 Hz EM noise coming from power lines. High- and low-pass filters had their half amplitude cut-off set at 0.01 and 100 Hz. Signals were digitized at 248 Hz.

### Data processing and measures

Electrophysiological data were processed with the EEGLAB and the ERPLAB toolboxes for MATLAB^85^. Large artifacts, such as blinks, eye movements and myograms, were removed using an independent component analysis (ICA)^86^. The infomax algorithm ICA was performed on a copy of the continuous EEG that was high-pass filtered at 1 Hz and low-pass filtered at 30 Hz. The resulting ICA weight matrix and sphering matrix were then applied to the continuous 0.1–30 Hz filtered EEG by using the ICLabel EEGLAB extension to signal artifactual independent components (ICs) that had more than 20% of chance of being muscle activity or more than 8% of chance of being eye movements^87^. These ICs were then systematically subtracted from this continuous 0.1–30 Hz filtered EEG, as in Finke et al.^88^, Goregliad et al.^89^ and Markey et al.^90^.

Only trials including behavioral responses performed between 300 and 2500 ms post-onset were retained to eliminate trials to which participants did not pay enough attention to, were too hesitant, or provided rash responses. The EEG epochs for those retained trials were taken from 200 ms pre-stimulus to 1000 ms post-stimulus. Their baselines were set by computing the mean voltage in the −200 to 0 ms time window for each electrode and by subtracting this mean value from each point of the −200 to 1000 ms epoch. Trials with voltages which amplitude exceeded ±100 μV at one or more of the four frontal electrodes (Fp1/2, F7/8) or which exceeded ±75 μV at one or more of the other 24 electrode sites were rejected. Trials with one or more flat lines lasting longer than 100 ms were also excluded. Only participants having at least 30 accepted trials in each condition were kept. Out of the 192 participants, 17 were rejected. Table 3 provides, for the remaining 175 participants, the average number of accepted trials and the standardized measurement error (SME) for the N1, P2, N400 and LPP amplitudes in each condition across the remaining 175 participants.

**Table 3:** Average numbers of accepted- and of rejected-trials and mean amplitude of the N1, P2, N400 and LPP ERPs at Cz and mean amplitude of these ERPs averaged across the 28 electrodes.

		Low SPQ group		High SPQ group	
		Accepted	Rejected	Accepted	Rejected
Numbers of trials accepted	*M* (SD)	66.0 (25.4)	119.6 (29.3)	82.1 (29.5)	102.7 (29.2)
	Range	31.0–152.0	36.0–165.0	34.0–164.0	30.0–155.0
N1 at Cz (µV)	*M* (SD)	1.4 (2.6)	1.3 (2.4)	1.7 (2.6)	1.7 (2.7)
	Range	−3.7–10.7	−4.9–10.1	−4.5–8.8	−4.2–10.9
Averaged N1 at 28 electrodes (µV)	*M* (SD)	0.7 (2.1)	0.6 (2.0)	0.6 (2.1)	0.6 (2.2)
	Range	−10.3–10.7	−8.7–10.1	−12.2–8.8	−10.6–10.9
P2 at Cz (µV)	*M* (SD)	1.7 (2.6)	1.6 (2.7)	2.8 (3.1)	2.6 (3.0)
	Range	−3.7–8.3	−5.1–9.1	−3.5–11.1	−3.4–12.3
Averaged P2 at 28 electrodes (µV)	*M* (SD)	1.2 (2.4)	1.1 (2.3)	1.7 (2.6)	1.6 (2.5)
	Range	−11.5–10.9	−11.2–9.6	−8.4–11.5	−7.0–12.3
N400 at Cz (µV)	*M* (SD)	−1.8 (3.5)	−2.2 (3.5)	−0.6 (3.1)	−1.1 (3.0)
	Range	−11.4–6.9	−10.0–7.5	−9.3–7.1	−9.5–6.4
Averaged N400 at 28 electrodes (µV)	*M* (SD)	−0.3 (2.8)	−0.5 (2.8)	0.3 (2.5)	−0.1 (2.6)
	Range	−11.4–12.5	−10.7–11.9	−10.6–9.0	−9.5–9.4
LPP at Cz (µV)	*M* (SD)	1.3 (3.4)	0.7 (3.3)	1.0 (4.7)	1.1 (4.3)
	Range	−6.8–11.1	−6.9–9.7	−14.8–14.4	−11.9–10.6
Averaged LPP at 28 electrodes (µV)	*M* (SD)	1.3 (2.8)	1.0 (2.6)	1.0 (3.2)	1.1 (2.9)
	Range	−7.1–14.0	−7.0–13.1	−14.8–14.4	−11.9–10.6

ERPs at each electrode were calculated by averaging all the accepted EEG epochs of each role category and response type. The measures used were the mean voltages of the ERPs at each electrode in specific time-windows, namely in the 160 to 220 ms post-stimuli one for the N1, in the 220 to 280 ms one for the P2, in the 300 to 500 ms for the N400, and in the 600 to 1000 ms window for the LPP.

### Statistics

Participants were median split into low- and high-schizotypy subgroups using their total SPQ score. The percentage of the roles accepted in each of the conditions was calculated by dividing the number of accepted roles by the total number of roles that the participant responded to within that particular condition.

Pearson’s *r* correlation analyses were then used to explore the relationship between the global- and three-subscale SPQ scores and these acceptance percentages for each role condition. The same was done for the total PDI scores and the scores at each of its subscales. One-tailed tests were used since the a priori hypothesis as to the direction of the differences. Mixed-model repeated-measure ANOVAs were run to analyze the RTs, N1, P2, N400, and LPP measures (see Supplementary Materials for the choice of this type of analysis). In the RT analyses, decision (i.e., accept vs. reject) and social roles category (ordinary vs. extraordinary vs. favorable vs. unfavorable) were the within-subject factors and the SPQ was the between-subject variable. In the analysis of the ERPs, within-subject factors were either the category of social roles (i.e., ordinary vs. extraordinary) and electrode or decision and electrode. The SPQ (high vs. low) was always the between-subject variable. Given the right hemiscalp maximum of the SPQ effect observed on P2 amplitudes, two ANOVAs were added to see whether this lateralization was significant, one for parasagittal (Fp1/2, F3/4, Fc3/4, Cp3/4, P3/4, O1/2) and one for lateral (F7/8, Ft7/8, Tp7/8, T5/6) electrodes. Both ANOVAs had hemiscalp (left vs. right) and antero-posterior location as within-subjects factors.

The above analyses were completed with IBM SPSS Statistics (version 27). Degrees of freedom were adjusted with the Greenhouse and Geisser’s^91^ procedure to compensate for the heterogeneity of variances across electrodes. In those cases, the original *F* values and degrees of freedom are provided together with the corrected *p* values. Effect sizes were reported as the proportion of variance explained by the variable (*ηp*^2^). The Benjamini-Hochberg^92^ false discovery rate (B-H FDR) procedure was then used to judge the results of each series of tests. *p* values were thus ranked from the most to the least significant. One B-H FDR threshold for each of these *p* values was then computed by dividing its rank by the total number of tests and by multiplying the result by the false discovery rate chosen (i.e., 10%). The *p* value was declared significant if it was smaller than that threshold, which was indicated by adding a star to the *p* (i.e., p*) in the “Result” section.

### Supplementary information

Supplementary Materials 1 (stimulus timing information)

Supplementary Materials 2 (list of the names of social roles used and characteristics)

## Data Availability

All datasets (behavioral and EEG) can be obtained from the corresponding author upon request.

## Code availability

The code for the stimulus sequence, EEG and ERPs processing and statistical analyses is available upon request to the corresponding author.
