# Supplementary material for: Finding normal-to-better neurocognitive indexes in individuals with schizotypal traits using a social role task
Source: Schizophrenia (Heidelb). 2023 Sep 29;9(1):66. doi: 10.1038/s41537-023-00394-5 (PMC10541438; doi:10.1038/s41537-023-00394-5)
Supplement: Supplementary file 1 — Supplementary Materials 1 (stimulus timing information) [file 41537_2023_394_MOESM1_ESM.docx]

**Supplementary Materials**

1. Stimulus paradigms

Eighty-four participants went through paradigm one (see Fig. 1, below). There, each role was presented for 500 ms, followed immediately by a fixation cross, which lasted for a randomized duration comprise between1500 to 2500 ms. Every 5 roles, the fixation cross was replaced by a ‘BLINK!’ stimulus, which lasted for 500 ms.

Thirty-seven participants went through paradigm two (Fig. 2, below). There, each role was presented for 1800 ms, immediately followed by a ‘BLINK!’ stimulus, which lasted for 1000 ms, and was then replaced by a fixation cross, which lasted for a randomized duration comprised between 300 to 1000 ms.

Fifty-four participants went through paradigm 3 (Fig. 3, below). Each role was presented for 1800 ms, immediately followed by a ‘BLINK!’ stimulus, which lasted for 500 ms and was then replaced by a fixation cross, which lasted for a randomized duration comprised between 800 to 1500 ms.


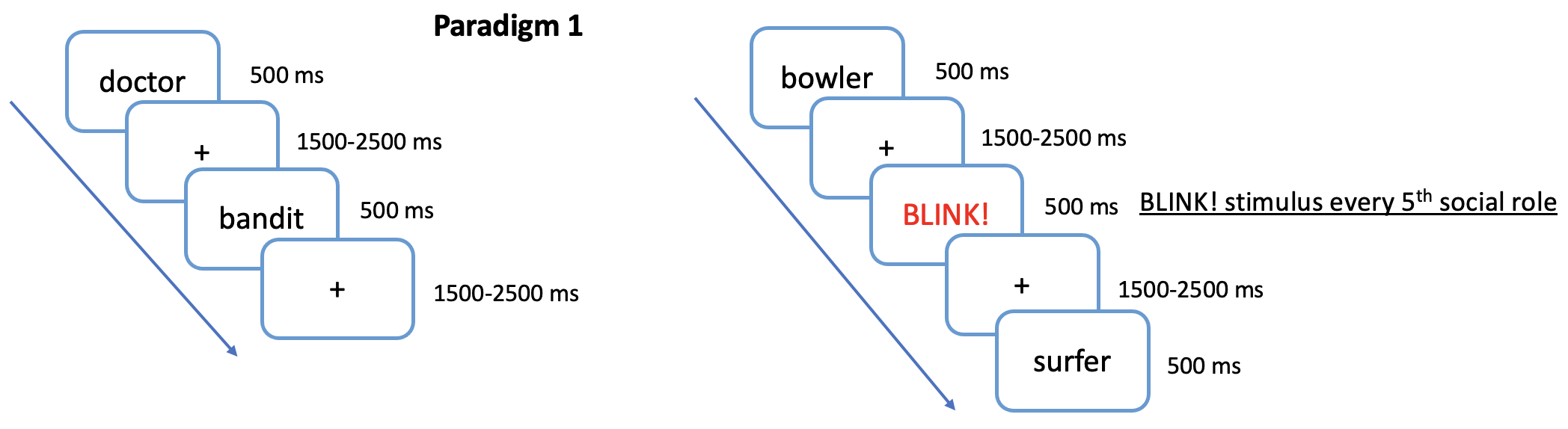


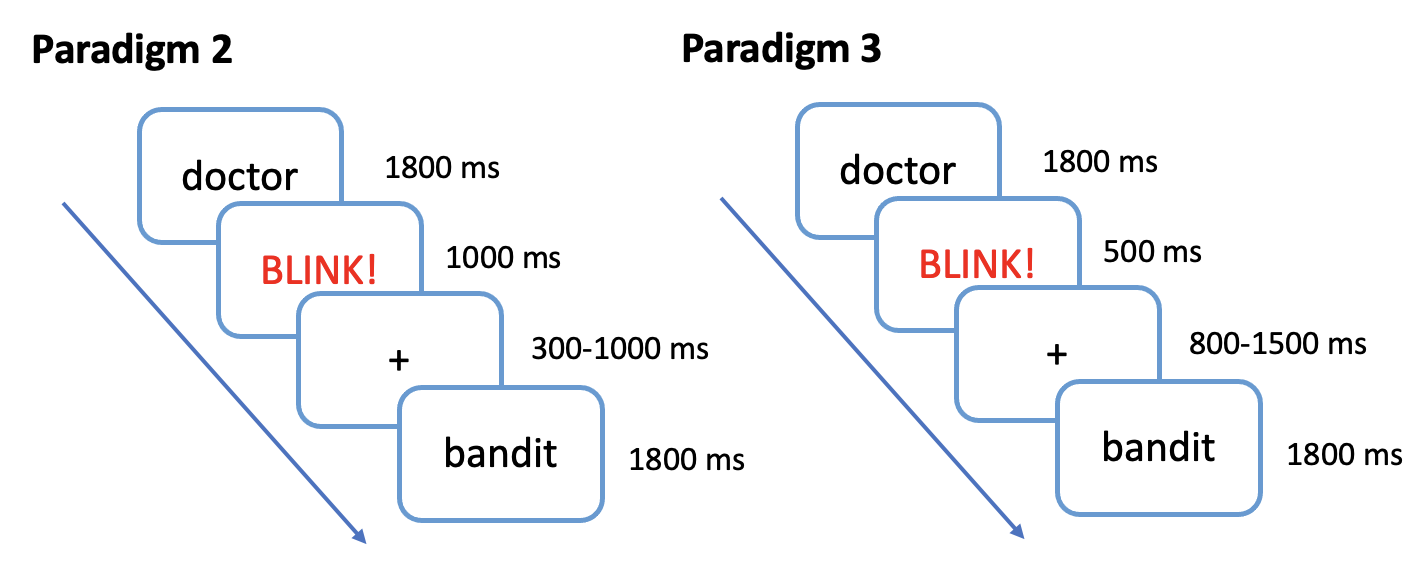


1. The choice of the frequentist approach

Frequency ANOVAs were chosen rather than the Bayesian approach because the main purpose of the study was to test null hypotheses, that is, to replicate the absence of longer reaction time in high- than in low-SPQ participants and the absence of smaller ERPs.

1. Supplementary Tables of the omnibus ANOVA and of the Benjamini-Hochberg FDR procedure on the N400 mean amplitudes at all electrodes.

Table a. Results of the ANOVA on N400 mean amplitudes over decision condition

| Number of tests (N) | Factors  SPQ (SPQ, 2 levels)  Decision (Dec, 2 levels)  Electrode (E, 2 levels) | df | F-values | p-values (Greenhouse-Geisser) | Significant according to B-H | Effect size (ηp^2^) | Observed Power (alpha=0.05) |
| --- | --- | --- | --- | --- | --- | --- | --- |
| 7 | SPQ | 1,173 | 3.37 | 0.068 | Yes | 0.019 | 0.45 |
|  | Dec | 1,173 | 23.07 | 3.0x10^-6^ | Yes | 0.12 | 1.00 |
|  | E | 27,4671 | 36.24 | 4.9x10^-23^ | Yes | 0.17 | 1.00 |
|  | SPQ x Dec | 1,173 | 0.98 | 0.324 | No | 0.006 | 0.17 |
|  | SPQ x E | 27,4671 | 4.28 | 0.004 | Yes | 0.024 | 0.89 |
|  | Dec x E | 27,4671 | 2.83 | 0.014 | Yes | 0.016 | 0.85 |
|  | SPQ x Dec x E | 27,4671 | 1.18 | 0.315 | No | 0.007 | 0.43 |

Table b. Results of the ANOVA on N400 mean amplitudes over category condition

| Number of tests (N) | Factors  SPQ (SPQ, 2 levels)  Category (Cat, 2 levels)  Electrode (E, 2 levels) | df | F-values | p-values (Greenhouse-Geisser) | Significant according to B-H | Effect size (ηp^2^) | Observed Power (alpha=0.05) |
| --- | --- | --- | --- | --- | --- | --- | --- |
| 7 | SPQ | 1,173 | 3.82 | 0.052 | Yes | 0.022 | 0.49 |
|  | Cat | 1,173 | 7.85 | 0.006 | Yes | 0.043 | 0.80 |
|  | E | 27,4671 | 37.28 | 1.8x10^-23^ | Yes | 0.18 | 1.00 |
|  | SPQ x Cat | 1,173 | 1.55 | 0.215 | No | 0.009 | 0.24 |
|  | SPQ x E | 27,4671 | 4.38 | 0.004 | Yes | 0.025 | 0.89 |
|  | Cat x E | 27,4671 | 2.20 | 0.058 | Yes | 0.013 | 0.70 |
|  | SPQ x Cat x E | 27,4671 | 1.38 | 0.234 | No | 0.008 | 0.47 |
